# Collecting and Preserving Marine and Freshwater Isopoda (Crustacea: Peracarida)

**DOI:** 10.3897/BDJ.3.e4912

**Published:** 2015-05-12

**Authors:** Regina Wetzer

**Affiliations:** ‡ Natural History Museum of Los Angeles County, Los Angeles, United States of America

**Keywords:** habitat, trap, night light, net, sieve, dredge, box core, ethanol, storage

## Abstract

**Background:**

Isopoda are the most diverse Crustacea. In order to encourage the study of isopod crustaceans and their use in biodiversity studies, systematics, ecology, physiology and more, one needs to know who the isopods are and where to find them.

**New information:**

This is a short “how to” guide focusing on the free-living marine and freshwater isopods: where they live and how to collect and preserve them. The tools and techniques described here are simple, but invaluable in accessing the natural history of these remarkable creatures.

## Introduction

In terms of body shape, size (0.5 – 500 mm), and the habitats in which they occur, the Isopoda are the most diverse Crustacea. They inhabit shallow water and live in the deep-sea. Some are pelagic, while others occur in interstitial spaces between grains of sand. Most are free-living, others are temporary or permanent parasites. Some live on land in leaf litter, others in underground waters, anchialine caves, wells, or thermal springs, and a few are even adapted to life in deserts. Approximately half of all isopod species live on land; the other half are aquatic. Of the aquatic species roughly 6,250 are marine ([Bibr B1561456][Bibr B1561466], Wetzer et al. 2013, Boyko et al. 2012, Boyko et al. 2013, Smit et al. 2014) and 500 species are associated with freshwater. With approximately 10,000 described species and a fossil record dating back to at least the Carboniferous (300 mya), isopod diversity and persistence is truly remarkable.

Eleven suborders are recognized. The Cymothoida and Sphaeromatidea are the replacement taxa for the paraphyletic Flabellifera (see Brandt and Poore 2003, Wetzer et al. 2013). The Cymothoida are most diverse in the tropics, with most members of the plesiomorphic and speciose family Cirolanidae living as active predators. Derived families (e.g., Aegidae, Cymothoidae, Corallanidae, Tridentellidae, Gnathiidae) are often temporary or permanent ectoparasites and micro-predators on fishes and other crustaceans. Anthuroidea are marine free-living or tube-dwelling, long, slender isopods commonly inhabiting offshore, soft-bottom environments. The Epicaridea, now recognized as two superfamilies Bopyroidea and Cryptoniscoidea, are exclusively parasites on other crustaceans (see Boyko et al. 2013).

The Sphaeromatoidea includes the speciose family Sphaeromatidae, the families Serolidae, Basserolidae, Plakarthriidae, Bathynatallidae, the *incerta sedis* genus *Paravireia*, and the extinct Schweglerellidae. The closely related Valvifera and Sphaeromatoidea are benthic herbivores common on marine shorelines worldwide whose pleopods are restricted to a branchial chamber for respiration rather than both locomotion and respiration as in most Cymothoida. A few sphaeromatids occur in freshwater. All Valvifera are marine. The Limnoriidea (families Limnoriidae, Keuphyliidae, and Hadromastacidae) are herbivores with the largest family, the Limnoriidae, predominately tropical borers of wood, mangroves, and other hard substrates. The sole species of Phoratopidea is marine, rare, and we know little about its ecology.

The Asellota occur in both freshwater and marine environments and are the predominant deep-sea (>500 m) isopod taxon. They have radiated widely, with some re-emerging into shallower water, while others occur in freshwater habitats including caves and ground water. Microcerberidea inhabit coastal ground waters or are interstitial. Calabozoida are known only from freshwater springs in Venezuela and Brazilian karst. Tainisopidea occur in freshwater relictual habitats. Although the Phreatoicidea fossil record indicates a globally-distributed marine past, today all Phreatoicidea species occur exclusively in freshwater of Australia, Tasmania, New Zealand, South Africa, and India. Phreatoicidea occur in groundwater habitats, ponds, pools, moist puddles, occasionally burrows, mosses, and moist habitats with very little water. The Oniscidea have diversified based on the isopod attribute of direct development (there are no free-living larval stages in isopods except in Epicaridea), and they are fully terrestrial. Insects aside, oniscids (also known as rolly polies, pill bugs, sow bugs, and woodlice) are the most diverse crustacean group to invade land. Collecting techniques applicable to oniscids are more similar to those employed by entomologists than those used by marine biologists and are not further addressed here.

## Discussion

**Habitats**

Most isopods hide, presumably to avoid predation. The iconic *Bathynomus*, at nearly a half meter long, may hold its own, although healed scars on museum specimens indicate they too sometimes tangle with predators. Only a few species, such as the pelagic species of *Anuropus* (Anuropidae) and some mid-water asellotans, occur in open water. Some Cirolanidae, Corallanidae, and occasionally juvenile Aegidae and Cymothoidae can occur in the water column and can be lured to light or baited traps.

Most isopods live in benthic habitats under rocks, in rock crevices and rock rubble, and amongst rocks and shells previously bored by sponges, mollusks, and worms. They are commonly found in coral rubble, dead coral heads, coarse shelly sand, and loose conglomerate rubble. Some live in algal holdfasts or on algal blades and stipes. Others occur in algal turf, sea grasses, gorgonians, and the interstices of live sponges. They can inhabit dead barnacle tests, abandoned worm tubes, and even the tunics of tunicates. Some marine asellotans, microcerberids, and a few sphaeromatids are so small they can be considered meiofaunal. Limnorids and some sphaeromatids bore holes into mangrove prop roots, kelp stipes, dead logs and tree stumps, wooden pier pilings and boats, and mud and rock banks.

Cymothoids, aegiids, tridentellids, corallanids, gnathiids, and some cirolanids are temporary or permanent parasites or micro-predators on the skin of fish with some species having specific body preferences. Some cymothoids exclusively inhabit the mouths of fish, while others exist only in the gill chambers. Some cymothoids and aegiids are associated with specific fish species, others have less specific host requirements. Some cirolanids are extreme scavengers and in large numbers can denude caged fish and dead humans of flesh in a very short time, functioning as the marine equivalent of dermestid beetles.

Some gnathiids (juvenile praniza life stage) are readily collected from fish gills and external body surfaces; adult gnathiids are free-living. Anthurids commonly live in tubes or burrows and within holdfasts and in rubble and so are rarely collected in large numbers. Aquatic Phreatoicidea live in freshwater springs and underground aquifers, but some occur in roadside ditches, cow patties, and crayfish burrows. Freshwater sphaeromatids, cirolanids, and asellotans occur in ground water, springs, and caves.

Most marine isopod taxa are well represented in the tropics where they also achieve their greatest diversity. Valviferans are the exception, as they are more diverse in the temperate regions of the world. Serolids are most speciose in the cold Southern Hemisphere marine environments, especially in the waters surrounding Antarctica. In general, species of all isopod taxa are larger in temperate and polar regions and smaller in the tropics.

There are few habitats isopods have not exploited. Few isopods inhabit mud flats with fine silty sediments. They are seldom found in disturbed habitats or in poorly circulating water. They never occur in temporary ponds. They are also rare in live coral. Once removed from their natural habitats many succumb quickly as they have difficulty keeping debris from fouling their pleopods and bodies. Maintaining marine and freshwater isopods, although successfully accomplished by some, is usually not a lackadaisical affair and requires well established and maintained aquaria. *Sphaeroma serratum* is an exception to this rule. It lives in mud burrows and uses the long setae on its pereopods to collect bacteria and detritus. The author has successfully maintained these in the lab in a collecting tray covered with only moistened newspaper covering the mud for months.

Isopod families and some large genera are largely ubiquitous with very low endemism. Isopod species, unlike amphipods and some other peracarids, have high regional endemicity and hence some previously understudied areas are full of new species. Isopods inhabit the deep-sea, intertidal rocky shores, algal beds, coral reefs, sand beaches, and subtidal sediments. Some are symbionts – both parasitic and not parasitic. Significant habitats include coral rubble, off-shore and inner-reef carbonate substrates which have very rich isopod faunas. Particularily well-sorted and well-aerated mobile sands especially around the bases of rocky shores and coral-reef outcrops can be hugely rich. Generally, they like well aerated water, and they don't like silt. The implications are that lagoons, estuaries, and mangroves have lower diversity than outer slope habitats.

**Collecting**

Intertidal and subtidal marine and freshwater collecting techniques employ similar equipment. Long and arduous hikes with heavy gear across reef flats or knife-edged rocky shores aside, it is easier to work in intertidal habitats compared to SCUBA diving, as one can cover much more "collecting" ground above water than underwater.

It is also nearly impossible to gauge the success of collecting while diving, whereas above water it is more likely that you will actually see your target as you are collecting it. Since isopod habitat preferences are diverse and many species have extremely patchy distributions, the need to cover considerable ground and sort through large enough samples should be taken into account. A penchant for selecting good isopod habitat comes with experience, practice, and specific knowledge about the locality.

**Figure 1.**
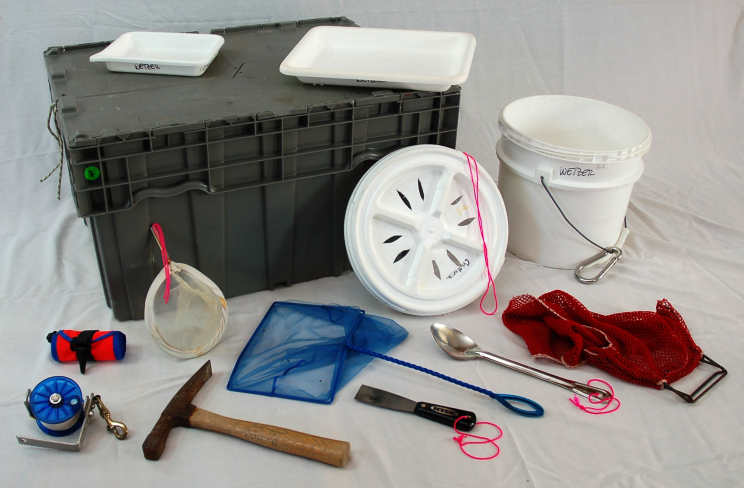
Storage and shipping box also holds dive gear on small boats, large and small photographic trays, 10-12 liter [3 gallon] bucket with modified screw top lid and stainless steel carabiner, mesh dive sack, aquarium fish net, brass-framed mermaid’s bra net (240 µm mesh or 63 µm for meiofauna), geology hammer, paint scraper, stainless steel spoon, dive sausage and reel. Photo credit: N. D. Pentcheff.

**Figure 2.**
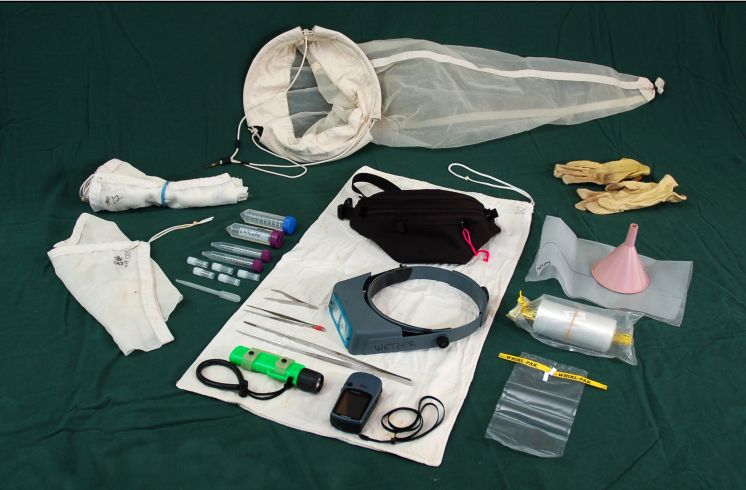
Plankton net (240 µm mesh), 25 x 25 cm [10 x 10 inches] 240 µm mesh plankton netting to use with funnel, numbered mesh draw-string collecting bags (28 x 46 cm [11 x 18 inches], 200 or 300 µm mesh, seams reinforced with sew-on edge binding), draw-string cotton sack 41 x 76 cm [16 x 30 inches] for larger bulkier samples, assorted screw-top vials and plastic eye dropper, stainless steel forceps (10 and 25 cm [6 and 10 inches] long), insect collecting forceps for picking up fast moving or fragile animals, leather garden gloves, Optivisor optical glass binocular magnifier (2 diopter, 1.5x), dive light, whirl top bags (2 sizes = 13 x 30 cm [5 x 12 inches] and 12 x 23 cm [4.5 x 9 inches]), fanny pack, and GPS. Photo credit: N. D. Pentcheff.

A 10-12 liter (three-gallon) bucket with screw top lid is indispensable (Fig. 1). These are sturdy, they nest well, and they are not too heavy to carry when they are full of rock, rubble, and water. They can serve as containers for collecting gear, and subsequently they are used for unsorted specimens, washes and rinses; they can even serve as a stool after a long day to take the weight off one’s feet. Numbered draw-string mesh sacks (~23 cm [9 inch] opening and roughly 30 cm [12 inches] tall made of 240 µm mesh) are invaluable for smaller samples (Fig. 2). Mesh sacks are preferred over plastic bags for most situations because water can be readily replaced, keeping invertebrates healthy and alive for much longer, especially in hot environments. The numbered sacks make it easy to keep track of specific habitats and samples. Field notes are recorded with #2 pencil on a dive slate or waterproof paper notebook. A geology hammer is useful for breaking apart porous rock and coral rubble, and a stout broad-bladed paint scraper can be used to remove dead barnacle tests, sponges, holdfasts, and other substrates. Some collectors include a dive knife in their repertoire. For intertidal collecting a small fanny pack is extremely useful for containing small items such as forceps, small pre-cut pieces of label paper, pencils, plastic tubes, and vials (some prefilled with alcohol). A small waterproof bag for the GPS and camera is helpful. Photographic trays are excellent for spreading out small samples for quick sorts and to determine whether one has selected the right substrate to collect the isopods of choice.

For SCUBA collecting the standard mesh dive bag is used to contain mesh sacks (described above), small plastic bags, plastic screw top tubes pre-filled with water (to keep them from floating away), leather garden gloves, and the geology hammer. When diving from an anchored boat in shallow water, pre-deploying several 10-12 liter [three gallon] buckets and attaching them with stainless steel carabiners at the base of the anchor chain is useful. Habitat samples are collected and contained in the same numbered mesh sacks described above (so that the infauna and your target isopods can’t get away). Plastic Ziplock bags are preferred over mesh bags for sand when SCUBA diving. Take <1 liter sand sample, hand-scooped into the bag. Sand compacts in larger bags and too much sand in buckets crushes specimens. "Coarse sand" such as shell grit (i.e., not really sand) is fine in bags and buckets.

At the end of the dive, the filled mesh sacks are placed in the buckets and tied off so they can’t fall out, and a diver’s buoy is inflated and sent to the surface to signal that the buckets can be pulled up. At the surface the water can be drained, making it easier to get the buckets into the boat, and the buckets can be refilled once on board. Lift bags can be used to get larger, heavier samples to the surface. Small plastic bags are used for individual specimens or very small samples. Always take a GPS reading before departing your dive site.

**Figure 3.**
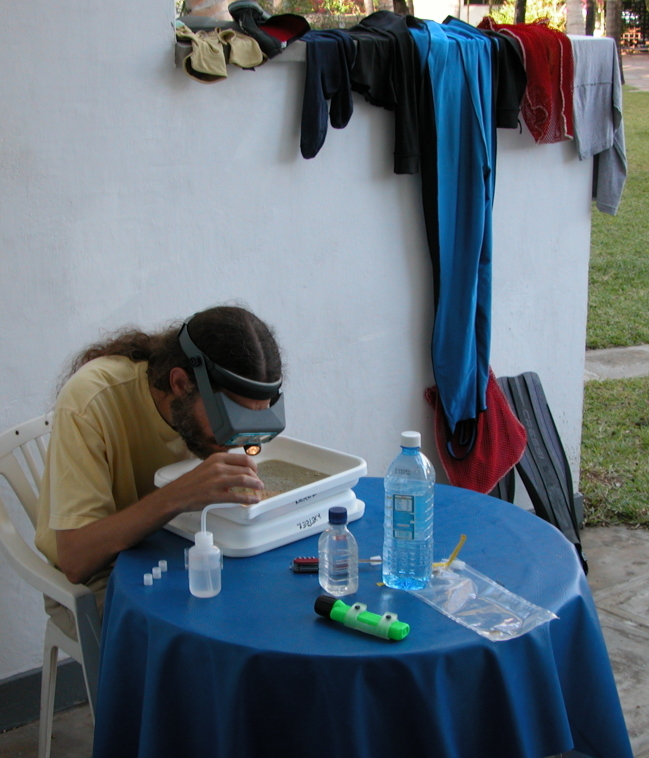
N. Dean Pentcheff removing live isopod specimens from a coral rubble washed sample.

**Specimen washes and concentrating sample:** Once you have collected a specific habitat, the next step is to remove the isopods. Since they are almost always hiding in complex substrate, removing at least a reasonable number by washing and concentrating the sample is preferred over sorting the entire sample, since only rarely does field time permit this luxury. Live sorting is always preferred over sorting preserved material (Fig. 3). Removing isopods from their substrate while still alive and then preserving them in vials of alcohol causes less damage to the specimens. This is especially important for fragile isopods like asellotans in order to get specimens with intact uropods, legs, and antennae. Individually preserved isopods are also less fouled by flocculants that settle out variously in bulk samples. If the collected material cannot be sorted in the time available, the only choice is to preserve the collection to be sorted later. Rinsing unsorted marine collections with freshwater before preserving with alcohol is preferred over not rinsing specimens. Removing the salt reduces the amount of flocculant settling out on especially setose specimens. If freshwater is not available, preserving directly with alcohol is the next best choice.

Once bulk samples are collected the next challenge is separating isopods from their substrate by irritating clinging organisms enough that they let go of the substrate. To do this, add ~60 – 125 ml of 95% ethanol to the bucket containing the unsorted substrate and water. Stir gently with a large stainless-steel serving spoon and let stand for a few minutes. If you can’t spare the ethanol, replace about 1/3 to 1/2 of the seawater in the bucket with freshwater.

If you are working in the tropics, use leather gloves (encounters with fire worms, stomatopods, and moray eel bits are not pleasant). If the sample contains sponges, use stout latex or plastic waterproof gloves. If you are not initially especially sensitive, you will be after a week in the field. If you are doing a moderate amount of processing, you will have swollen unhappy hands from the spicules and toxins released by some of the sponges and algae; gloves help prevent or ameliorate this.

The preferred method is elutriating with a sea-water hose at the base of the bucket. Such an arrangement is commonly only available on board a ship or well outfitted marine lab. A modified version of this technique is to proceed by taking a small handful of algae or rubble and swishing it through the water, shaking off isopods and other invertebrates inside the bucket. If the rubble or rocks are large, break them up first with the hammer. Move each washed handful of substrate to a second bucket. Proceed until the large pieces are removed.

**Figure 4.**
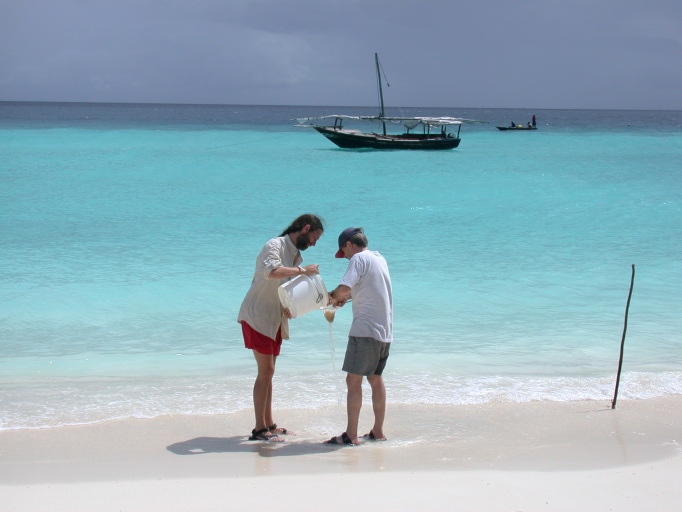
N. Dean Pentcheff and Niel Bruce straining seawater containing isopods into mermaid's bra (netting). The bucket is swirled to suspend animals and quickly strained over the netting to retain them.

With most of the free-living invertebrates remaining behind in the water, swirl the bucket and strain the water through a 240µm mermaid’s bra (Fig. 1 and Fig. 4). A piece of plankton netting folded into quarters and bent open to make a funnel will also suffice. Rest the netting on a plastic or metal funnel held over the second bucket containing the substrate you removed previously. Strain the water and specimens onto the netting. Invert the mermaid’s bra or netting and back-wash specimens into a petri dish or photographic tray. Wash rubble or algae at least twice gently. Too much washing results in specimens being beaten up; not enough washing and the animals don’t come off. Be sure to check the bottom of the bucket. Heavy animals like crabs, mollusks, and large sphaeromatids have a tendency to sink to the bottom. This method will remove most animals (isopods, other peracarids, worms, small mollusks, shrimps, etc.). Cirolanids, aegids, tridentellids, and corallanids are more resilient than sphaeromatids, anthurids, and asellotans, and they cling much more tightly to the substrate and may need additional coaxing with freshwater to be removed. Scan the sand left in the bottom of the bucket or in a photo tray with magnifying glasses to catch any large-bodied specimens. Asellotans, anthurids, gnathids, sphaeromatids and others will have washed off in the earlier washes.

If working in the tropics be careful to keep samples out of the sun and heat. Tropical sun and heat quickly kill invertebrates. Ascidians, sponges, and algae will quickly ruin good samples with nasty, stinky slime and muck that does not easily wash off specimens.

The more fresh water you have the better. If you have no fresh water, do the best you can by rinsing specimens with fresh, clean seawater. You may have to clean rare and valuable specimens gently in an ultrasound back in the lab, but that is still better than having no specimens at all.

**Baited traps and night lights:** Commercial traps of various sizes are available and have been described by others (e.g., Keable 1995, Manning 1986). A simple, multi-purpose baited trap or light trap can be made by using the aforementioned versatile 10-12 liter [3-gallon] bucket. To make a simple baited trap, place your bait of choice: oily fish such as sardines, herrings, mackeral, tuna and a small dive weight in the bottom of the bucket. Always use fresh bait. If that is not possible, tinned sardines, crabs, and molluscs are better than no bait). To make a light trap use a cyalume stick instead of bait. Screw on the lid, which has had holes cut into it (see Fig. 1). Attach the handle to a rope and tie it off on a pier or dock after dark. If on a reef flat, wade out to the desired depth and set on the bottom securely. Setting traps from a boat is done by setting a strong line to the float and "a weak" line fixing the trap to the weight so when the weight is fouled, one can recover the trap. Record GPS position. Pour your drink of choice and enjoy it for about an hour. The amount of time a trap should be deployed varies based on habitat and local environmental conditions, but usually not longer than overnight. Leaving the trap *in situ* too long will attract larger predators that may eat your isopods. Retrieve the bucket and concentrate the catch through a mermaid’s bra or plankton net. The advantage of collections made with traps is that specimens do not need to be separated from their substrate.

Similarly, a weighted plankton net can be used as a light trap by affixing a dive light near the net opening. Hold the plankton net in place for a few minutes and then periodically sweeping it through the water column to collect the animals attracted by the light. This works best in shallow water as the isopods must swim to reach the light. If the distance between the net and their source is too large, the isopods can become someone’s meal before they reach the net.

A one liter soda bottle can easily be turned into a single-use disposable baited trap, see Manning (1986) for details. These traps are usually best left out overnight. It is best to place them within a larger scampi, shrimp, crab or lobster trap which reduces trap loss to large fish such a sharks. Disposable plastic bottle traps sometimes do not prevent fish, crabs, and mollusks from breaking in and stealing the bait and destroying the device. They are inexpensive. Make several.

**Collecting by dredging and box core methods:** These techniques are commonly used for asellotans, serolids, and sometimes cirolanids. Small hand-held dredges are sometimes used while SCUBA diving, but small sample sizes make them less effective than other methods. Methods targeting other invertebrates (e.g., mollusks) will on occasion collect isopods too, but usually only in small numbers. Epibenthic sleds are the most productive tool for collecting from below SCUBA depths to the deep sea. Sleds are designed with stabilizing planes to keep the sled from sinking too deep into the mud or soft bottom. In contrast dredges are designed to bring up mud or soft bottom by scooping or dragging the apparatus across the bottom. Most sleds are operated from a boat or ship. Some good sledding can be done from small boats to about 50m using a small Ockleman sled. Specimen catches can be improved with a couple of torches (dive lights) in the mouth of the sled. Some hand-held epibenthic sleds are available, but a simple, effective, and inexpensive one can be made from a dust pan with the back cut off and a net attached. These can be used while SCUBA diving as one swims along, skimming the pan over the sand, with one hand stirring up the sediment ahead of the sled. In a pinch the plankton net works too, its circular opening is not quite as efficient as the rectangular hand dredge.

**Collecting from sand and pore water:** Sandy beaches and river and stream edges are commonly sampled along a transect from the high tide mark to the low tide line by sieving sand through a sturdy sieve. For collecting phreatoicids in their natural terrestrial habitats or in disturbed environments (e.g., cow pastures and roadside ditches), a sturdy plastic kitchen sieve to separate animals from water and muck works well. Small interstitial sand dwellers (microcerberids, some sphaeromatids) are extracted by digging a hole and letting the surrounding water fill it in (Chappuis 1942, Hunt and Stanley 2000). Isopods usually start swimming and even small individuals are readily seen and netted. Sweep a 50–63 µm mesh sieve or net through the water to collect isopods washed out from among the sand grains. If you do not have a net, use a wide mouthed container to scoop out the water. Concentrate the sample over 50–63 µm mesh plankton netting. Suction sampling devices for meso- and microfauna (up to 200 µm) are described by Delamare-Deboutteville (1960), Bou and Rouch (1967), Tabacchi (1990); these methods are commonly used by meiofauna specialists.

**Collecting in caves and other cryptic habitats:** Removing fragile, individual specimens from silty environments such as marine caves is accomplished with a “Sket bottle”. This tool is also effective for collecting from rubble filled bottoms, branching corals, small holes, and cracks on rocky surfaces. Chevaldonné et al. (2008) do a wonderful job describing the manufacture and use of this device. For larger specimens (e.g., large phreatoicid species living in crayfish burrows) a yabby pump is used.

**Collecting fish ectoparasites and micropredators:** Aegids, cymothoids, gnathids, and sometimes corallanids are attached to the body surface, gills, or in the mouths of fishes. Parasites are symbionts that spend most of their lives attached to fish, whereas micro-predators are analagous to mosquitos, genuine blood and tissue feeders, that attach to hosts for an extended period. After feeding micro-predators drop off and spend time hiding in the sediment. Often ectoparasites are firmly attached, and a piece of the fish must be removed to avoid damaging the isopod parasite. Fishermen, fish taxonomists, fish parasitologists, and fish markets are always excellent resources for larger fish parasites. Commonly micro-spears while SCUBA diving and rotenone (a non-selective piscicide) are used to collect individual fish. Throw-netting for shallow schooling fish can also be productive. Micro-predatory isopods (e.g., gnathids) are commonly collected by fish seining with each fish immediately put in a plastic bag before being brought to the surface. At the surface the fish is released in a bucket of seawater, and the areas (body, gills) with attached parasites are gently massaged until the parasites release. The water containing the parasites is sieved to concentrate the sample.

**Preservation:** Unsorted material as well as sorted specimens should be preserved directly in 95% not denatured ethanol in a volume roughly 1:3 (specimen to ethanol). Absolute ethanol is not favored, as it contains additional hydrocarbon dehydrating agents to increase the alcohol percentage. Since specimens are already in water, 5% more water is not a problem, and 95% ethanol is much cheaper than absolute ethanol. If high quality not denatured 95% ethanol is unavailable, the next best choice is rum, vodka, tequila, or other local available liquor with the highest possible alcohol content (see Wall et al. 2014, Tang 2006).

Upon return to the home laboratory, samples are always rinsed once more with freshwater run over a plankton net and the ethanol is replaced. Museum-quality printed specimen labels are inserted into vials and jars with specimens and lots are stored in museum jars. Specimens maintained at low temperature (refrigerated), then preserved with 95% ethanol while still alive, yield excellent material for DNA extraction and amplification. Poor extraction and failed amplifications result from allowing samples to putrefy in the field and from inadequate presevation in the field. The smaller the specimen, the more important it is to pay attention to proper preservation.

Not only does this preservation method yield excellent results for later molecular work, it also works well for morphological specimens and scanning electron microscopy. For morphology, ethanol concentration can be reduced to 70-80% for long term storage. Preserving isopods directly in RNAlater (trademark of Ambion, Inc., Austin, Texas) or freezing them with liquid nitrogen yields the best material for DNA extraction with both bountiful and high quality DNA. Access to and transport of liquid nitrogen may not be practical. RNAlater is expensive, and most of us can only afford to fix small lots of specimens in this way. Although not ideal, RNAlater-preserved specimens can also be used for morphology by thoroughly and repeatedly rinsing specimens in distilled water and subsequently transferring them to 75-95% ethanol.

During much of the 20th century, formalin was advocated as a fixative. We now know that formalin badly damages DNA, making formalin fixation highly undesirable (Tang 2006). Using alcohol (or salt solutions such as RNAlater) avoids having to sequester formalin-contaminated utensils and equipment and ensures that specimens will retain their complete scientific value.

**Figure 5.**
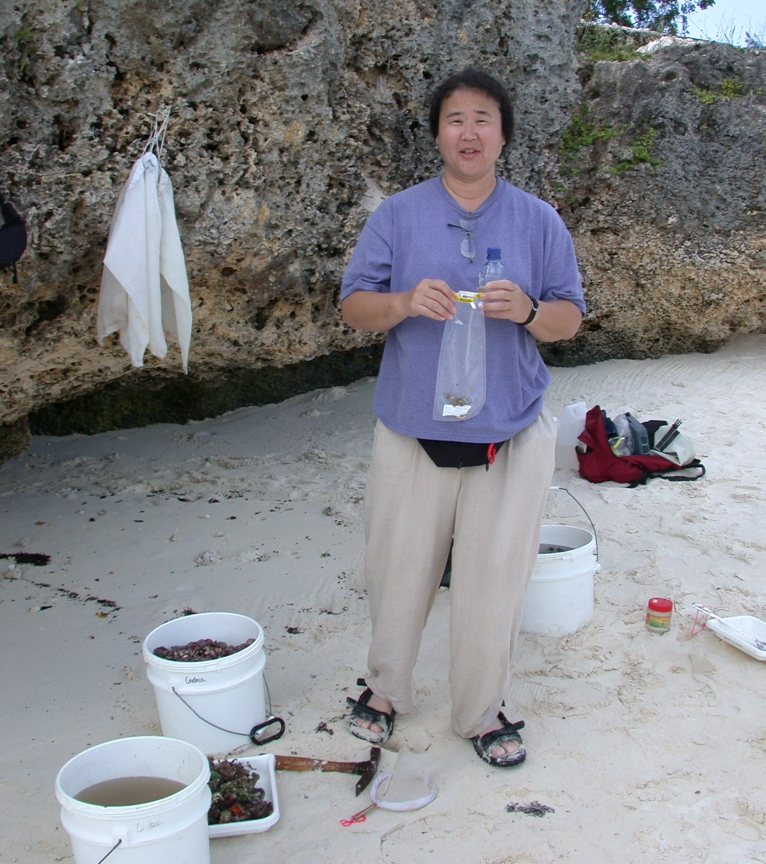
Kathy Omura holding whirl top bag containing sample which is ready to be filled with ethanol.

**Temporary specimen storage:** Plastic screw cap vials for individual specimens and small lots work well and reduce the amount of ethanol needed (Fig. 2 and Fig. 5). Combining multiple vials in whirl top (also known as whirl pack) bags is helpful. Unsorted samples destined to be sorted back home are transferred to a whirl top bag with a spoon. Residual sample that is stuck to the bucket, tray, or petri dish is washed into a mermaid’s bra. The mermaid’s bra is inverted over a funnel held over the opening of the whirl top bag and washed into the bag with ethanol from a squeeze bottle. The bag is filled with ethanol (1:3 volume as described above), the air is squeezed out of the bag, and the top of the bag is rolled down to the alcohol level and the ends are tied. A second whirl top bag is slipped over the first bag and the specimen label facing out is slipped between the two bags. The air is squeezed out, and again the top is rolled down and the ends are tied. When tying whirl top bag closures make sure the wire ends are not placed where they might puncture the sample bag.

**Field data:** Enter collecting and locality data into a laptop computer or other electronic device nightly. Back it up, and if possible transfer your data the cloud or your home institution. Periodically photograph the field notebook in case you loose the laptop. Avoid putting labels or debris in directly with specimens as it can abrade specimens and inadvertently break off delicate legs, antennae, and uropods.

**Back in the laboratory:** To unpack the sample back at the lab, use a pair of scissors to cut off the top of the outer whirltop bag. Remove the inner bag, unroll its top, and use scissors to cut off the top. Pour the bag contents into a mermaid’s bra, rinse with fresh water, and transfer sample to specimen jar with label. Refill the jar with new alcohol. Plastic (disposable) pipettes come in handy for sucking up small specimens and a spoon works well for larger ones, making transfer of specimens to vials and bags efficient.

**Collecting Permits:** Make sure you have the proper collecting permits and know the legalities of transporting specimens across national and international borders. Be aware that applying for and securing the proper national and local collecting permits may take many months and in some cases permits need to be initiated at least a year in advance. Sometimes permits may also be expensive and involve police checks, a research visa, and other administrative necessities.

**“Do little harm:”** Collecting isopods and their bi-catch ultimately means killing them and disturbing their habitat. Be mindful of your impact and always minimize the number of specimens you take and maximize their ultimate use. Properly curate your specimens (proper and complete labeling, museum grade specimen jars for long-term storage, highest grade ethanol). Contact a major natural history museum if in doubt about best practices. Deposit your properly curated specimens for long-term storage in an accredited natural history museum. Consult the Society for the Preservation of Natural History Collections (www.spnhc.org/) for the latest best practices.

## Supplementary Material

Supplementary material 1 United States vendors for commonly used supplies. Data type: list
File: oo_39110.docx Wetzer
