# Supplementary material for: Collecting and Preserving Marine and Freshwater Isopoda (Crustacea: Peracarida)
Source: Biodivers Data J. 2015 May 12;(3):e4912. doi: 10.3897/BDJ.3.e4912 (PMC4442250; doi:10.3897/BDJ.3.e4912)
Supplement: Supplementary material 1 — United States vendors for commonly used supplies. [file biodiversity_data_journal-3-e4912-s001.docx]

Aquatic Eco-Systems, Inc. [http://www.aquaticeco.com]
2395 Apopka Blvd., Apopka, FL 32703: mesh filter sacks (bags)

Consolidated Plastics Company, Inc. [http://www.consolidatedplastics.com]
8181 Darrow Rd., Twinsburg, OH 44087: storage tote with hinged lid, plastic bucket (pale) with screw on lid

Fine Science Tools [http://www.finescience.com/]
373-G Vintage Park Drive, Foster City, CA 94404-1139: forceps

Forestry Suppliers, Inc. [http://www.forestry-suppliers.com]
205 West Rankin Street, P.O. Box 8397, Jackson, MS 39284-8397: Rite in the Rain waterproof notebooks

Nasco Whirl-Pak Bags [http://www.enasco.com/page/wp_baginfo]
4825 Stoddard Road, P.O. Box 3837, Modesto, California 95352-3837: Whirl-Pak bags

Wildlife Supply Company [http://wildco.com/]
95 Botsford Place, Buffalo NY 14216: plankton nets
